# Parkinson's Disease–Associated Kinase PINK1 Regulates Miro Protein Level and Axonal Transport of Mitochondria

**DOI:** 10.1371/journal.pgen.1002537

**Published:** 2012-03-01

**Authors:** Song Liu, Tomoyo Sawada, Seongsoo Lee, Wendou Yu, George Silverio, Philomena Alapatt, Ivan Millan, Alice Shen, William Saxton, Tomoko Kanao, Ryosuke Takahashi, Nobutaka Hattori, Yuzuru Imai, Bingwei Lu

**Affiliations:** 1 Department of Pathology, Stanford University School of Medicine, Stanford, California, United States of America; 2 Department of Neurology, Graduate School of Medicine, Kyoto University, Kyoto, Japan; 3 CREST (Core Research for Evolutionary Science and Technology), Japan Science and Technology Agency, Saitama, Japan; 4 Neuroscience Program, Stanford University School of Medicine, Stanford, California, United States of America; 5 College of Arts and Sciences, Washington University, St. Louis, Missouri, United States of America; 6 Department of Molecular, Cell, and Developmental Biology, University of California Santa Cruz, Santa Cruz, California, United States of America; 7 Research Institute for Diseases of Old Age, Juntendo University Graduate School of Medicine, Tokyo, Japan; 8 Department of Neurology, Juntendo University Graduate School of Medicine, Tokyo, Japan; 9 Department of Neuroscience for Neurodegenerative Disorders, Juntendo University Graduate School of Medicine, Tokyo, Japan; The University of North Carolina at Chapel Hill, United States of America

## Abstract

Mutations in Pten-induced kinase 1 (PINK1) are linked to early-onset familial Parkinson's disease (FPD). PINK1 has previously been implicated in mitochondrial fission/fusion dynamics, quality control, and electron transport chain function. However, it is not clear how these processes are interconnected and whether they are sufficient to explain all aspects of PINK1 pathogenesis. Here we show that PINK1 also controls mitochondrial motility. In *Drosophila*, downregulation of dMiro or other components of the mitochondrial transport machinery rescued *dPINK1* mutant phenotypes in the muscle and dopaminergic (DA) neurons, whereas dMiro overexpression alone caused DA neuron loss. dMiro protein level was increased in *dPINK1* mutant but decreased in dPINK1 or dParkin overexpression conditions. In *Drosophila* larval motor neurons, overexpression of dPINK1 inhibited axonal mitochondria transport in both anterograde and retrograde directions, whereas dPINK1 knockdown promoted anterograde transport. In HeLa cells, overexpressed hPINK1 worked together with hParkin, another FPD gene, to regulate the ubiquitination and degradation of hMiro1 and hMiro2, apparently in a Ser-156 phosphorylation-independent manner. Also in HeLa cells, loss of hMiro promoted the perinuclear clustering of mitochondria and facilitated autophagy of damaged mitochondria, effects previously associated with activation of the PINK1/Parkin pathway. These newly identified functions of PINK1/Parkin and Miro in mitochondrial transport and mitophagy contribute to our understanding of the complex interplays in mitochondrial quality control that are critically involved in PD pathogenesis, and they may explain the peripheral neuropathy symptoms seen in some PD patients carrying particular *PINK1* or *Parkin* mutations. Moreover, the different effects of loss of PINK1 function on Miro protein level in *Drosophila* and mouse cells may offer one explanation of the distinct phenotypic manifestations of *PINK1* mutants in these two species.

## Author Summary

Parkinson's disease (PD) is the second most common neurodegenerative disease. It mainly affects movement in elderly people and was traditionally considered a sporadic disease with no known cause. Discoveries of genes associated with familial PD (FPD) have demonstrated that PD pathogenesis can be significantly influenced by an individual's genetic makeup. Understanding the functions of these FPD genes will allow better understanding of the sporadic PD cases. *PINK1* and *Parkin* are genes associated with FPD that affect patients at an early age. Mutations in PINK1 and Parkin lead to the accumulation of damaged mitochondria, the powerhouse of the cell, as a result of impairments of the mitochondrial quality control system. However, the mechanism of PINK1/Parkin action remains poorly understood. Here we show that PINK1 and Parkin act together to regulate Miro, a key component of the mitochondrial transport machinery, and that altered activities of PINK1 cause aberrant mitochondrial transport. Regulation of mitochondrial transport may be a critical aspect of the mechanisms by which the PINK1/Parkin pathway governs mitochondrial quality control. Dysfunction of this process could contribute to the loss of DA neurons, the cardinal feature of PD, as well as the peripheral neuropathy symptom associated with particular *PINK1* or *Parkin* mutations.

## Introduction

PD is a neurodegenerative disorder characterized by the dysfunction and loss of dopaminergic (DA) neurons in the substantia nigra, although neurons in other brain regions are affected as well. Mutations in *PINK1* and *Parkin* are linked to familial forms of early-onset PD [1], [2]. *PINK1* encodes a Ser/Thr kinase with a mitochondrial targeting sequence, whereas Parkin encodes an E3 ubiquitin ligase. Studies in *Drosophila* first revealed that *PINK1* and *Parkin* act in a common pathway to impact mitochondrial function and DA neuron maintenance [3]–[6], in part through the regulation of mitochondrial fission/fusion dynamics [7]–[11]. At least in primary cultured mammalian hippocampal neurons and DA neurons, PINK1 and Parkin have been shown to exert similar effects on mitochondrial dynamics as seen in *Drosophila* DA neurons [7], [12]. PINK1 and Parkin are also implicated in mitochondrial quality control [13]. Decreased mitochondrial membrane potential stabilizes the normally labile PINK1, which recruits Parkin to damaged mitochondria, leading to ubiquitination of mitochondrial proteins and marking damaged mitochondria for removal by autophagy [14]. Both mitochondrial fission/fusion dynamics and autophagy are considered important aspects of the mitochondrial quality control mechanism that mediates PINK1/Parkin function in DA neuron maintenance [11], [15]–[17].

In some *PINK1*- or *Parkin*-linked PD patients, symptoms of peripheral neuropathy were also reported [18]–[20]. It is not clear whether this is caused by defects in the aforementioned functions or some other unknown function of PINK1/Parkin. Peripheral neuropathy is a clinical term used to describe various forms of damages to nerves of the peripheral nervous system by distinct mechanisms [21]. Many types of peripheral neuropathy are dependent on the length of neuronal axon, with neurons carrying long axons frequently affected. It is hypothesized that this is caused by defects in the axonal transport of key proteins and/or organelles such as mitochondria, which are critical for maintaining the axonal and synaptic physiology of those extremely polarized neurons [22]. This notion has gained significant support from recent studies of the inherited forms of peripheral neuropathies [22], [23]. Defective mitochondrial transport has also been considered a pathogenic event in other neurodegenerative diseases [22], [24], [25], including rodent models of PD [26], [27]. In primary cultured rat hippocampal neurons, overexpression of PINK1 has been shown to inhibit the lateral movement of photoactivated, mito-Dendra2-labelled mitochondria [12], raising the possibility that defects in the axonal transport of mitochondria may actively participate in PINK1-related PD pathogenesis.

A major aspect of axonal transport is mediated by motor proteins that travel on axonal microtubules, which are polarized and uniformly orientated, with their plus-ends pointing towards nerve terminals. The kinesin and dynein motors are involved in microtubule plus-end (anterograde) and minus-end directed (retrograde) transport, respectively [28]. Mitochondria are mainly produced in neuronal cell body and delivered to sites where metabolic demand is high, such as the synapses and nodes of Ranvier [29]. The functions of Mitochondrial Rho (Miro), a mitochondrial outer membrane GTPase [30], and the cytosolic protein Milton are critical for mitochondrial transport, as they serve to link mitochondria with kinesin motors and the microtubule cytoskeleton [31], [32]. In *Drosophila* Miro or Milton mutants, mitochondria accumulate in neuronal soma and fail to move into the axons [31], [32]. In cultured mammalian cells, overexpression of a constitutively active mutant of Miro was shown to induce cell death, suggesting that mitochondrial transport or some other aspect of Miro function is important for cell survival [30]. Whether this is relevant to *in vivo* conditions such as neurodegenerative disease settings is not known.

The fruit fly *Drosophila melanogaster* has served as an excellent model for studying neurodegenerative diseases [10]. It was in *Drosophila* that the *in vivo* function of PINK1 was first revealed [3]–[6]. PINK1 mutant flies exhibit abnormal wing postures, reduced flight ability and thoracic ATP level, degeneration of indirect flight muscle and DA neurons, and male sterility, which are caused by the accumulation of dysfunctional mitochondria, thus suggesting a role of PINK1 in mitochondrial function and/or quality control [3]–[6]. Further genetic studies in *Drosophila* have also uncovered important functions of PINK1 in regulating mitochondrial morphology and electron transport chain activity [7]–[9], [33].

The power of the *Drosophila* neurodegenerative disease models lies in the ability to facilitate unbiased genetic modifier screens to identify new players involved in the disease process. Using this approach, we show in this study that PINK1 genetically interacts with the mitochondrial transport machinery. Reduction of function in Miro, Milton, or kinesin heavy chain effectively rescued the *PINK1* mutant phenotypes. On the other hand, overexpression (OE) of Miro led to the formation of enlarged mitochondria and resulted in DA neuron loss, thus phenocopying *PINK1* mutants. By monitoring mitochondrial movement in live *Drosophila* larval motor neurons, which possesses long axons and could serve as a model system for studying peripheral neuropathy, we provide evidence that PINK1 directly regulates mitochondrial transport. The function of PINK1 in mitochondrial transport may contribute to PD pathogenesis in DA neurons and underlie the peripheral neuropathy symptoms associated with certain *PINK1* mutations in some PD patients.

Our biochemical analysis demonstrated that overexpressed PINK1 in cooperation with Parkin could regulate Miro protein ubiquitination and stability, which might contribute to the regulatory effect of PINK1 on mitochondrial motility. While our paper was under review, it was suggested that PINK1 phosphorylates Miro at a conserved S156 residue, and that this phosphorylation event is required to activate proteasomal degradation of Miro in a Parkin-dependent manner [34]. However, our *in vitro* kinase assay using active recombinant PINK1 failed to show direct phosphorylation of Miro by PINK1. Moreover, a mutant form of hMiro1 with the S156 site mutated to Ala was equally susceptible to PINK1/Parkin-mediated degradation in HeLa cells. Thus, the exact molecular mechanism by which the PINK1/Parkin pathway regulates Miro protein level will require further investigation.

## Results

### Genetic interaction between PINK1 and the mitochondrial transport machinery

**Figure 1:**
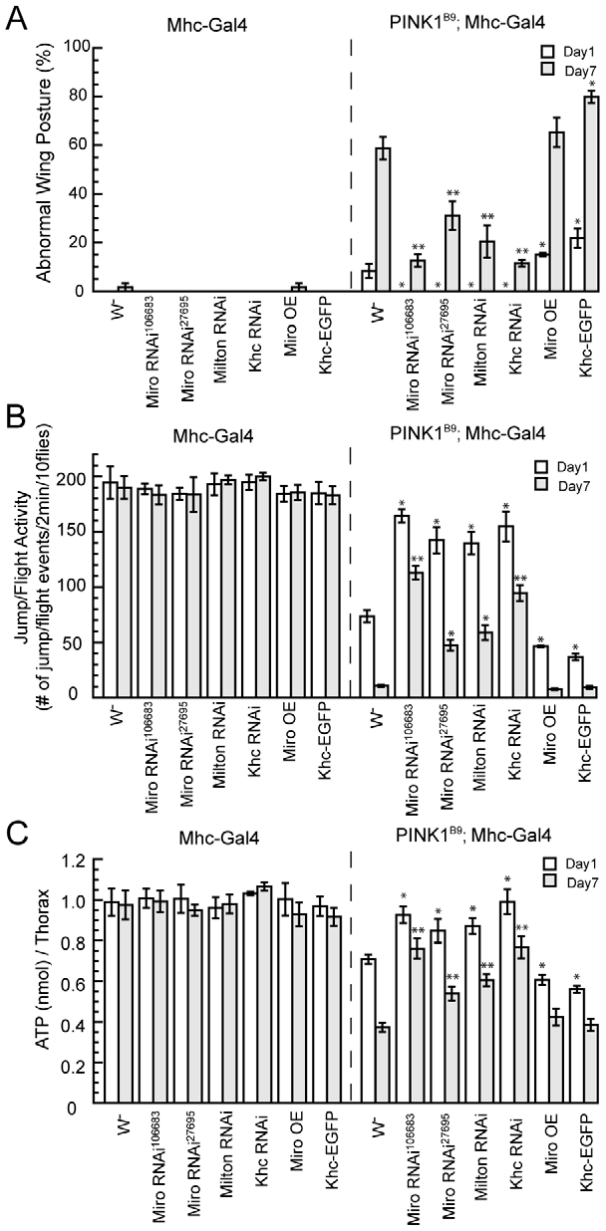
Genetic interaction between PINK1 and the mitochondrial transport machinery in the muscle. Effects of knockdown or overexpression of Miro, Milton or Khc on the abnormal wing posture (A), jump/fly ability (B), and thoracic ATP level (C) of *PINK1^B9^* mutant are shown. Male flies of the corresponding genotypes were scored at 1-day or 7-day of age at 25°C. Miro-RNAi^106683^ was used for the rest of studies. Data are from three independent experiments and the results are presented as mean ± s.e.m.

By taking advantage of the easily identifiable phenotype of abnormal wing posture induced by *dPINK1* inactivation, we performed a genetic screen for modifiers of *PINK1*. The scheme was similar as described before [35]. In this screen, we identified components of the mitochondrial transport machinery as genetic modifier of *PINK1*. Knockdown of *Miro*, *Milton* or Kinesin heavy chain (*Khc*) each rescued the muscle phenotypes in *PINK1^B9^* null mutant, including abnormal wing posture, decreased fly ability and ATP depletion (Figure 1A–1C). Conversely, overexpression (OE) of Miro and Khc enhanced such phenotypes (Figure 1A–1C). These results demonstrated strong genetic interaction between PINK1 and the mitochondrial transport machinery as a whole, supporting that mitochondrial transport is the underlying mechanism mediating their genetic interaction. In this study, we will focus our analysis on Miro, as a previous study in cultured cells suggested that mammalian Miro might physically interact with PINK1 [36].

### Genetic interaction between Miro and PINK1 in the PD–relevant DA neurons

**Figure 2:**
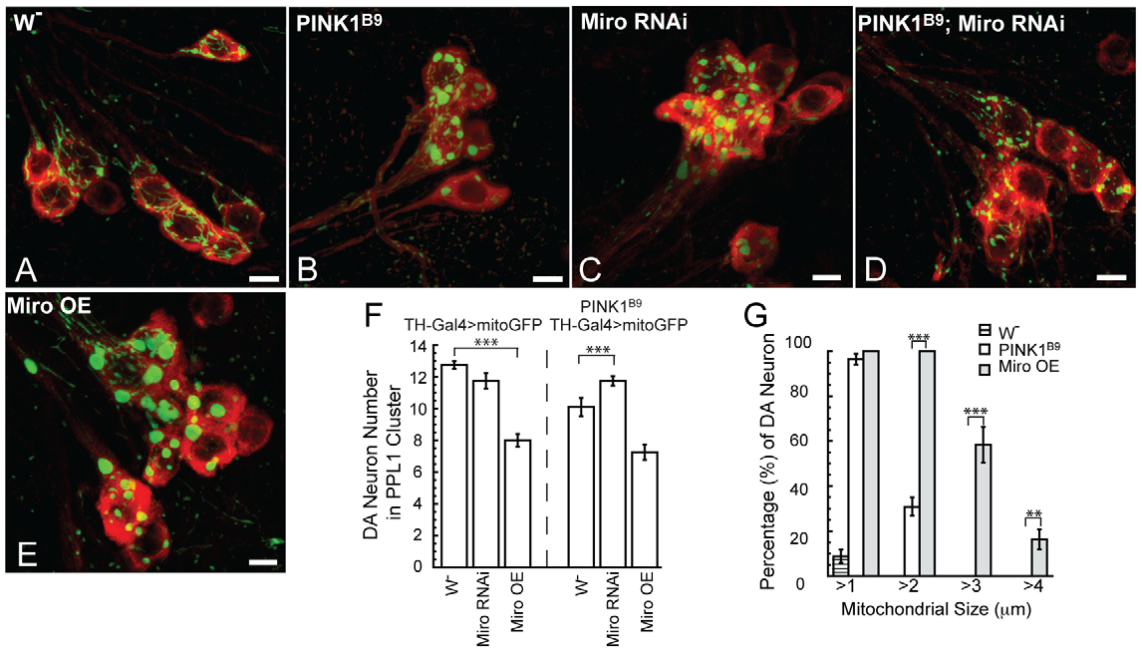
Genetic interaction between PINK1 and Miro in DA neurons. (A–E) Mitochondrial morphology in DA neurons of different genotyped flies visualized with mitoGFP expressed under *TH-Gal4* control. Red: TH, green: GFP. DA neurons of the protocerebral posterior lateral 1 (PPL1) cluster were shown. Scale bar: 5 µm. (F) Effects of Miro-RNAi and Miro-OE on DA neuron maintenance in wild type and *PINK1^B9^* mutant backgrounds. DA neuron number was scored in 14-day old flies. At least 5 flies were used for each genotype. (G) Mitochondrial-size distribution in DA neurons of the various genotyped flies. Data are presented as mean ± s.e.m.

To test the relevance of the functional interaction between PINK1 and the mitochondrial transport machinery to PD pathogenesis, we examined their interaction in DA neurons, the disease-relevant cell type. As in the muscle, Miro-RNAi effectively rescued *PINK1* mutant phenotypes in DA neurons, both in terms of mitochondrial aggregation (Figure 2A–2D) and DA neuron loss (Figure 2F). Moreover, Miro-OE alone, driven by the *TH-Gal4* driver, caused aberrant mitochondrial aggregation (2E, 2G) and DA neuron loss (Figure 2F), thus phenocopying PINK1 loss-of-function effects (Figure 2B, 2F, 2G), although the Miro-OE effect was noticeably stronger than *PINK1* mutant. It is worth noting that *TH-Gal4*-driven Miro-OE in *PINK1* mutant background resulted in dramatically reduced viability (data not shown), although the surviving adults did not show further DA neuron loss than that induced by Miro-OE alone (Figure 2F). Together, these results demonstrate that PINK1 and Miro also exhibit strong genetic interaction in DA neurons, with decreased dMiro level/activity ameliorating the detrimental effects caused by the loss of dPINK1, whereas increased dMiro level or activity phenocopying *dPINK1* mutants.

### PINK1 regulates mitochondrial motility in *Drosophila* larval motor neurons

**Figure 3:**
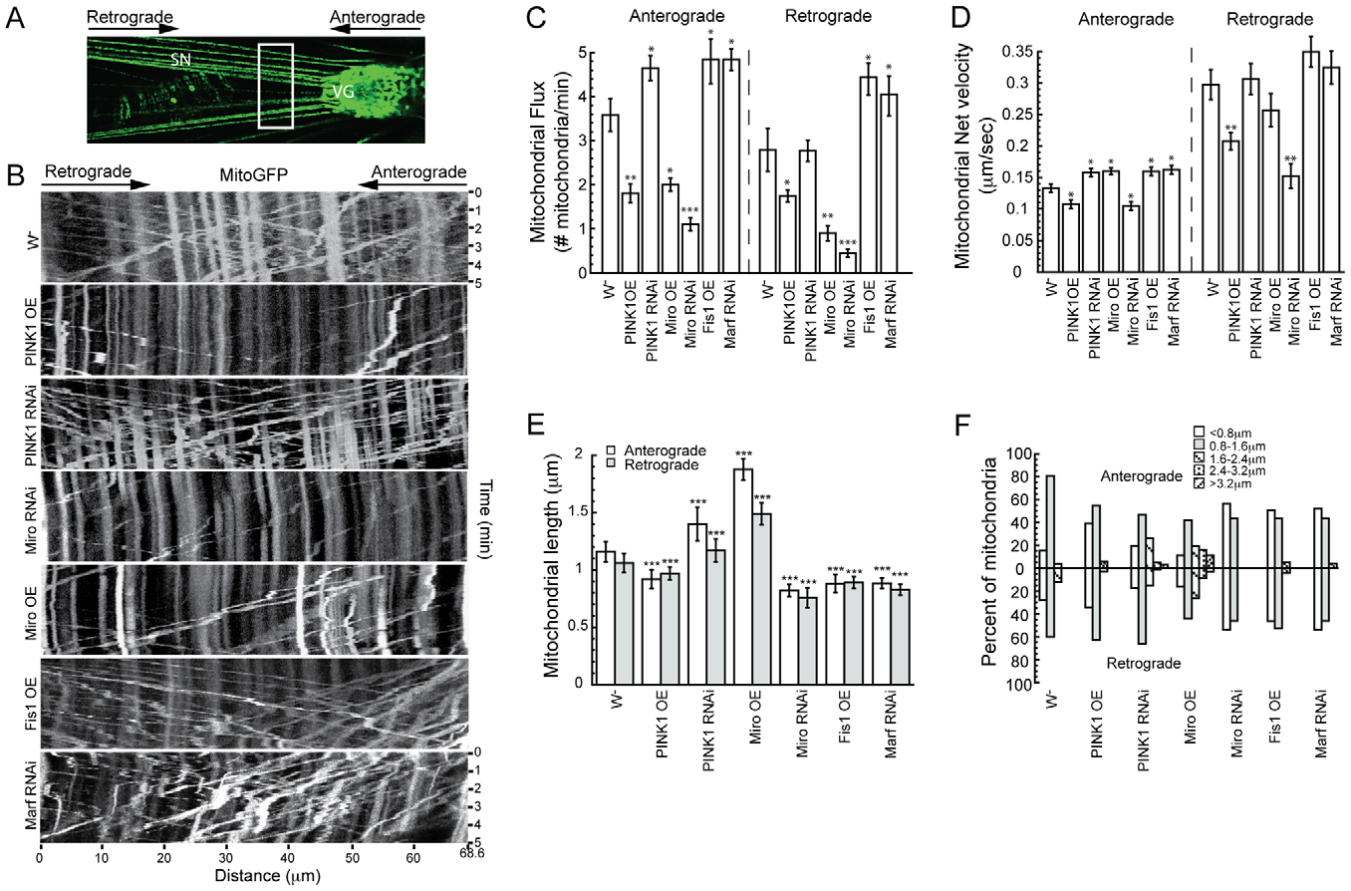
PINK1 regulates mitochondrial motility in *Drosophila* larval motor neurons. (A) An image of a third instar larva expressing mitoGFP in the motor neurons. For time-lapse imaging, mitochondrial movement in segmental nerves (SN) was recorded near the ventral ganglion (VG) (indicated by the white box). (B) Representative kymographs of mitochondrial movement in the different genetic backgrounds. Each panel is a kymograph representation of the position of fluorescently labeled mitochondria as a function of time. Anterograde mitochondrial movements have positive slopes, whereas retrograde mitochondrial movements have negative slopes. Stationary mitochondria appear as vertical streaks. (C, D) Mitochondrial flux (C) and net velocity (D) in different genetic backgrounds. (E, F) Average mitochondrial length (E) and mitochondrial length distribution (F) in the various genetic backgrounds. Data are presented as mean ± s.e.m.

The strong genetic interaction between PINK1 and Miro raised the interesting possibility that PINK1 might directly regulate mitochondrial transport, the impairment of which might contribute to PINK1-related parkinsonism. This was further supported by the DA neuron loss induced by Miro-OE alone, which presumably acted by altering mitochondrial transport. To test this idea, we examined the effect of PINK1 on mitochondrial movement in *Drosophila* larval motor neurons, a system amenable to live imaging of mitochondrial transport. Mitochondrially-targeted GFP (mitoGFP) expressed specifically in motor neurons was used to track mitochondrial movement via live imaging in anesthetized third instar larvae (Figure 3A), using well-established procedures [37], [38]. To highlight the mitochondria undergoing active transport, a 61.5 µm-long segment of motor neuron was photobleached and the movement of fluorescently labeled mitochondria moving into the bleached area from both directions was recorded at 1 frame/2 s for 300 s (Figure 3B, Videos S1, S2, S3, S4, S5, S6, S7). From these videos, mitochondrial flux (the normalized number of mitochondria that passes certain point over time), mitochondrial net velocity (the normalized mitochondrial net displacement over time), and mitochondrial morphology (e.g. mitochondrial length) in different genetic backgrounds were analyzed. In general, mitochondrial net velocity is controlled primarily by the intrinsic properties and quantities of motor proteins associated with the mitochondria [38], while mitochondrial flux can also be significantly affected by mitochondrial morphology, as changes in mitochondrial morphological features such as length can increase or decrease the number of motile mitochondria.

We found that PINK1-OE decreased mitochondrial flux as well as net velocity in both anterograde and retrograde directions, similar to the effect of Miro-RNAi, although the PINK1-OE effect appeared to be slightly weaker (Figure 3B–3D; Videos S1, S2, S4). In contrast, PINK1-RNAi and Miro-OE both increased the net velocity of anterograde mitochondrial transport, with retrograde transport largely unaffected (Figure 3B, 3C; Videos S3, S5). PINK1-RNAi also increased anterograde mitochondrial flux (Figure 3C), while mitochondrial flux in Miro-OE background was reduced in both anterograde and retrograde directions (Figure 3C). The reduction of mitochondrial flux by Miro-OE could be partially explained by the formation of very long mitochondria in Miro-OE motor neurons (Figure 3E, 3F), as previously observed [38].

In addition to mitochondrial motility, PINK1 also affected mitochondrial length in motor neurons. PINK1-RNAi increased mitochondrial length in the axons of larval motor neurons as in Miro-OE case, although the effect of Miro-OE was much stronger. Conversely, PINK1-OE and Miro-RNAi both decreased mitochondrial length (Figure 3E, 3F). To address whether PINK1-induced mitochondrial motility change was due to its effect on mitochondrial length, we examined mitochondrial transport in genetic backgrounds where mitochondrial fusion/fission machinery was directly manipulated to alter mitochondrial length. Increasing mitochondrial fission by overexpression of the fission protein Fis1 or knockdown of the fusion protein Marf led to decreased mitochondrial length (Figure 3E, 3F), similar to the effects of Miro-RNAi or PINK1-OE. However, in contrast to the decreased mitochondrial flux and net velocity as observed in the Miro-RNAi or PINK1-OE backgrounds, Fis1-OE and Marf-RNAi both increased mitochondrial flux and net velocity in anterograde and retrograde directions (Figure 3B–3D; Videos S6, S7), suggesting that mitochondrial length and transport kinetics are not always directly correlated, which is consistent with a previous report [38]. Collectively, these results support the notion that PINK1 regulates mitochondrial transport and that its effect on mitochondrial motility is direct, rather than a secondary effect of mitochondrial length change.

### Altered PINK1 activities affect mitochondrial distribution in motor neuron axons

**Figure 4:**
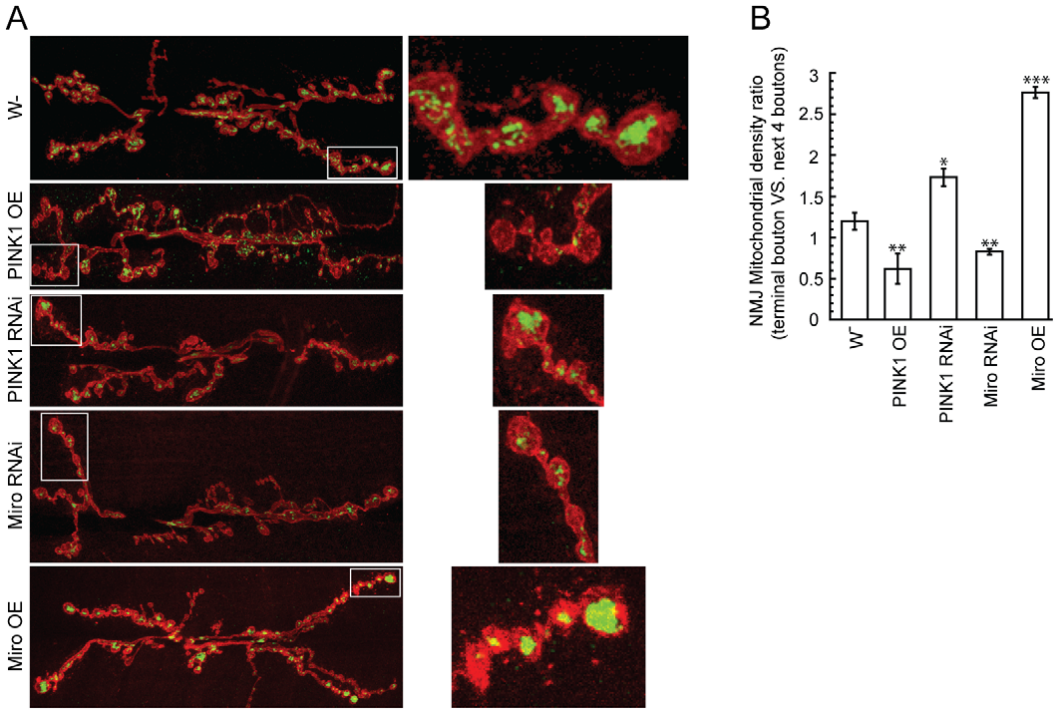
PINK1 affects mitochondrial distribution within motor neuron nerve terminals at the *Drosophila* larval neuromuscular junction. Pan-neuronal *elav-Gal4* driver was used to express mitoGFP in different genetic backgrounds to examine the mitochondrial distribution at the neuromuscular junction (NMJ) of muscle 6/7 in A3-A4 segments. Anti-GFP antibody (Green) and anti-HRP-Texas Red antibody (Red) were used to visualize mitochondria and boutons, respectively. (A) Representative images of mitochondrial distribution at the NMJs of wild type, PINK1-OE, PINK1-RNAi, Miro-RNAi and Miro-OE flies were shown in the left panels. Detailed mitochondrial distribution of the boxed areas in the left panels was shown in the right panels. (B) PINK1 regulates mitochondrial distribution at the NMJ. Mitochondrial density of each bouton was estimated by its average GFP fluorescence intensity, represented as total GFP green fluorescence (anti-GFP staining) of each bouton/bouton size (HRP-Texas Red staining). Mitochondrial density ratio of the most distal bouton versus the next 4 boutons was used to quantitatively compare mitochondrial distribution at the NMJ. Compared to the control, PINK1 knockdown showed significant accumulation of mitochondria at the most distal bouton, although its effect was not as strong as Miro-OE. In contrast, Miro-RNAi and PINK1-OE both led to reduced mitochondrial mass in the most distal boutons.

In addition to mitochondrial motility, we also examined mitochondrial distribution at motor neuron nerve terminals at the larval neuromuscular junction (NMJ), which can be used as an indirect measure of mitochondrial motility. Consistent with a previous report [31], Miro-OE led to the accumulation of mitochondria in the most distal boutons, which is likely the consequence of net anterograde transport (Figure 4A, 4B). PINK1 knockdown showed similar effect (Figure 4A, 4B). Thus, the dysfunctional mitochondria in *PINK1* mutant might gain longer retention time in the distal segment of motor neuron axons where synapses are formed. This finding may have clinical implications for PINK1 pathogenesis. In contrast, Miro-RNAi and PINK1-OE both led to decreased accumulation of mitochondria in the most distal boutons (Figure 4A, 4B). We conclude that PINK1 regulates mitochondrial distribution in motor neuron nerve terminals, likely through its effect on mitochondrial transport.

### Overactivation of the PINK1/Parkin pathway reduces Miro protein level

**Figure 5:**
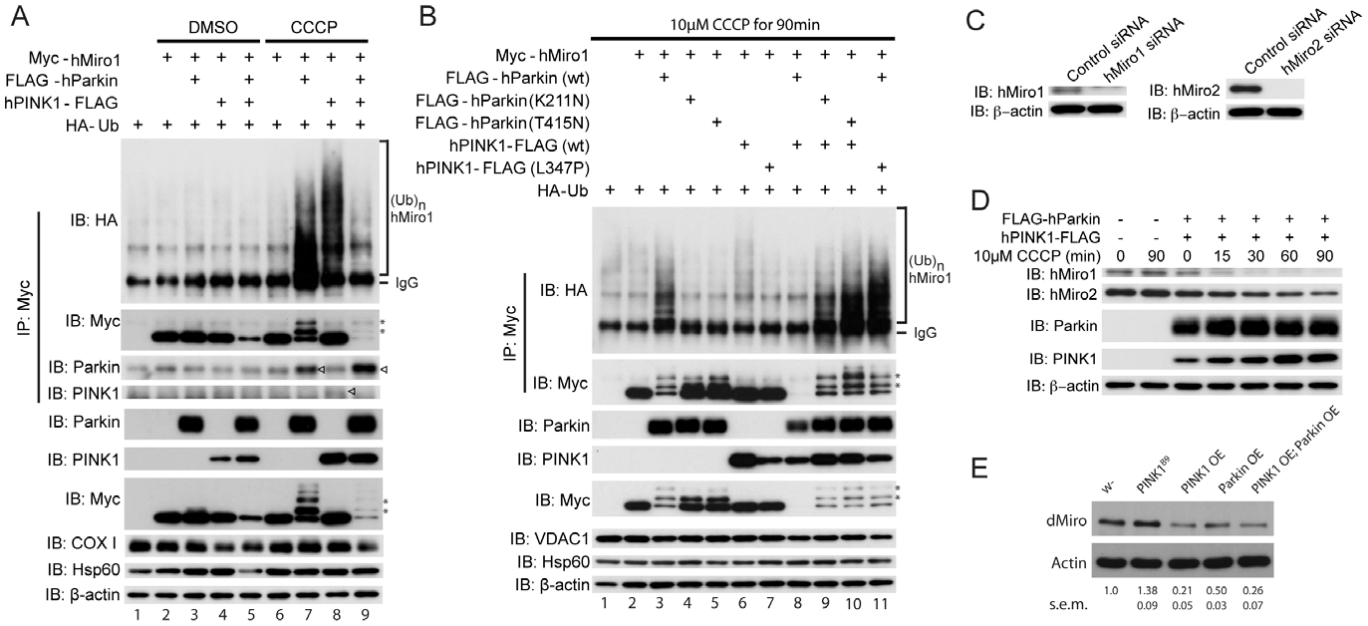
PINK1/Parkin regulates the ubiquitination and stability of Miro protein. (A) Effects of hPINK1 and hParkin on the ubiquitination and protein stability of exogenous hMiro1 in HeLa cells. Cells transfected with the indicated plasmids were treated with or without 10 µM CCCP for 90 min. Arrowheads, Parkin or PINK1 co-precipitated with hMiro1; Asterisks: oligo-ubiquitinated hMiro1. The inner mitochondrial membrane protein COX I and the mitochondrial matrix protein Hsp60 are used as loading controls for mitochondrial proteins. β-actin serves as a loading control for cytosolic proteins. (B) Effects of mutant hPINK1 or hParkin on hMiro1 protein level in HeLa cells. The Parkin K211N mutation in the linker domain is reported to lack both ubiqutin-ligase (E3) and mitochondrial translocation activities, while the Parkin T415 mutation in the RNIG2 domain, which disrupts E3 activity, retains partial mitochondrial translocation activity [56]. The PINK1 L347P mutant is reported to have reduced kinase activity [57]. An OMM protein VDAC1 is shown as a loading control for mitochondrial proteins. (C, D) Time-course experiments examining the effects of hPINK1/hParkin on the stability of endogenous hMiro1/2 proteins. Specificity of the hMiro antibodies was confirmed using hMiro knockdown cells (C). (E) Endogenous dMiro level in fly brain extracts of the various genetic backgrounds. *elav-Gal4* was used to drive the expression of the transgenes. Normalized dMiro level compared to wild type control was shown as mean ± s.e.m. (n = 3).

Our results so far showed that PINK1 and Miro exert opposite effects on mitochondrial morphology, motility and distribution. We next explored the biochemical mechanisms underlying their negative genetic relationship. We first used HeLa cells to test whether Miro protein level might be regulated by PINK1 and possibly Parkin, which tends to work together with PINK1 in a common pathway [3]–[5], [39]. There are two Miro homologues in human cells, hMiro1 and hMiro2, that are ∼60% identical [30]. Overexpression of either hPINK1 or hParkin did not lead to obvious change of exogenous hMiro1 protein level under normal conditions, but a modest reduction of hMiro1 level was observed when hPINK1 and hParkin were co-expressed (Figure 5A, lane 5). A decline in mitochondrial membrane-potential induced by the mitochondrial uncoupler carbonyl cyanide m-chlorophenylhydrazone (CCCP) was reported to activate the PINK1/Parkin pathway [13], [14], [39]. Under CCCP treatment condition, hPINK1 or hParkin each significantly stimulated hMiro1 ubiquitination (Figure 5A). Since HeLa cells express very little endogenous Parkin [13], the effect of hPINK1 alone on hMiro1 ubiquitination (Figure 5A, lane 8) suggested that other E3 ligase(s) might be recruited by hPINK1 to ubiquitinate hMiro1. However, this ubiquitination event did not appear to lead to destabilization of hMiro1 (Figure 5A, IB: Myc). In contrast, coexpression of hPINK1 and hParkin dramatically reduced hMiro1 level in the presence of CCCP (Figure 5A, lanes 9). Importantly, pathogenic mutations in hPINK1 or hParkin abolished this effect (Figure 5B, lanes 4 and 5 compared with lane 3, and lanes 9–11 compared with lane 8), indicating that functional hPINK1 and hParkin are both required in the destabilization of hMiro1. Previously, many outer mitochondrial membrane (OMM) proteins were shown to be degraded by the ubiquitin proteasome system (UPS) pathway in a PINK1/Parkin-dependent reaction at an early step of mitophagy, while other OMM proteins might be eliminated by subsequent autophagosome-dependent events [40], [41]. Thus, direct or indirect substrates of PINK1/Parkin could be distinguished by their degradation kinetics [40]. In our experiments, hMiro1 was more rapidly degraded than another OMM protein VDAC1 (Figure 5B, VDAC1), a reported Parkin substrate involved in mitophagy [14], supporting that hMiro1 is a direct substrate of Parkin.

Similar to hMiro1, hMiro2 could also be ubiquitinated by PINK1 and Parkin co-expression or after CCCP treatment. However, the degradation of hMiro2 was at a much slower rate compared to hMiro1 (Figure S1), consistent with a previous result [40]. Furthermore, like exogenous hMiro1, endogenous hMiro1 was also rapidly degraded by PINK1/Parkin overexpression in HeLa cells and its level was dramatically reduced within 15 min of CCCP treatment. The degradation of endogenous hMiro2 was again at a much slower rate than that of hMiro1 (Figure 5C, 5D).

We next examined the effect of the PINK1/Parkin pathway on Miro protein level in an *in vivo* setting. Similar to the results in HeLa cells, *Drosophila* dMiro protein level was decreased in the brain extracts of PINK1 or Parkin overexpression adult flies (Figure 5E). Conversely, dMiro level was increased in *PINK1^B9^* mutant brain extracts (Figure 5E). These results are consistent with dPINK1 negatively regulating dMiro protein level *in vivo*. It is worth noting that different from the effects seen in HeLa cells, overexpression of PINK1 or Parkin alone was sufficient to reduce dMiro level in adult *Drosophila* brain, and the co-expression of PINK1 and Parkin did not lead to much further reduction of dMiro level than PINK1-OE alone, suggesting that the endogenous levels or activities of PINK1 and Parkin are already sufficient to support each other's action in the *Drosophila* brain.

### Knockdown of Miro promotes the removal of damaged mitochondria by Parkin-mediated mitophagy

**Figure 6:**
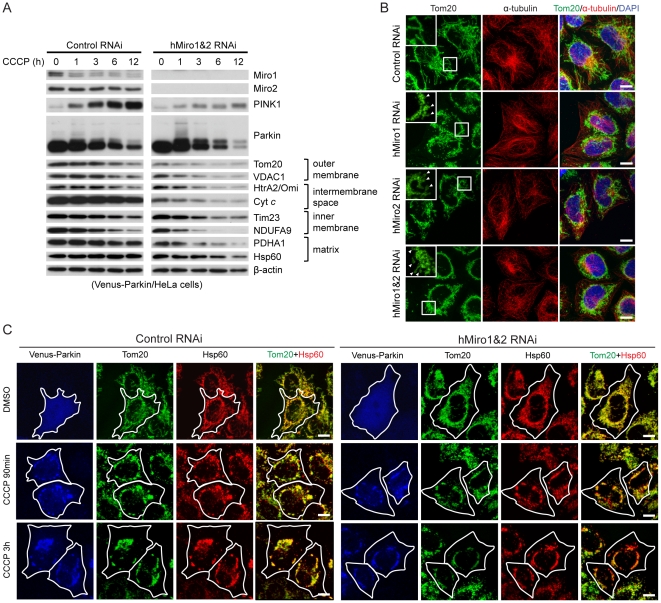
Miro knockdown facilitates the removal of damaged mitochondria by Parkin-mediated mitophagy. (A) Time course of CCCP treatment to monitor mitophagy in Hela cells stably transfected with Venus-Parkin. Cells were transfected with control siRNA or hMiro1+hMiro2 siRNAs and treated with 10 µM CCCP for the indicated time. Markers for mitochondrial outer membrane (Tom20, VDAC1), intermembrane space (HtrA2/Omi, Cyt *c*), inner membrane (Tim23, NDUFA9), and matrix (PDHA1, Hsp60) were examined by Western blot to determine the elimination of damaged mitochondria. β-actin was used as a loading control. (B) Immunofluorescence staining showing the formation of perinuclear mitochondrial aggregates after siRNA-mediated knockdown of hMiro1 or hMiro2. HeLa cells transfected with control siRNA, hMiro1 siRNA, hMiro2 siRNA, or hMiro1+hMiro2 siRNAs were stained for Tom20 and α-tubulin. Merged images are shown to the right. Insets show enlarged view of mitochondrial morphology in the boxed areas. Arrowheads indicate ring-like or round-shaped mitochondria. (C) Miro knockdown facilitates the early stage of mitophagy in venus-Parkin transfected HeLa cells treated with CCCP. HeLa cells stably transfected with venus-Parkin were co-transfected with control siRNA or hMiro1+hMiro2 siRNAs and then treated with the DMSO vehicle or CCCP for 90 mins or 3 hrs. Venus-Parkin (blue), Tom20 (green), and Hsp60 (red) were visualized by immunostaining. Merged Tom20/Hsp60 signals are shown to the right. Cells of interest are outlined.

Removal of damaged or dysfunctional mitochondria through mitophagy could be one mechanism by which the PINK1/Parkin pathway maintains mitochondrial health, at least under some conditions, and the accumulation of those abnormal mitochondria in *PINK1* mutants could be the underlying cause of disease pathogenesis. Consistent with this notion, it was previously shown that enhancing autophagy could efficiently rescue *dPINK1* mutant phenotypes [35]. To better understand the rescuing effect of Miro-RNAi in *PINK1* mutant background, we examined the effect of Miro knockdown on mitophagy. We used CCCP treatment to induce mitochondrial damage in HeLa cells stably transfected with venus-Parkin, and subsequently monitored the removal of damaged mitochondria over time by examining the protein levels of mitochondrial markers on the inner/outer membrane or in the matrix and inter-membrane space. Simultaneous knockdown of hMiro1 and hMiro2 significantly accelerated the mitochondrial removal process, with all the mitochondrial markers disappearing faster in hMiro knockdown cells than in the control siRNA-treated cells (Figure 6A). This suggested that there was more active mitophagy after hMiro knockdown. To confirm this result, we monitored the mitochondrial network by immunofluorescence staining. Compared to the control siRNA-treated cells, knockdown of either hMiro1 or hMiro2 led to the accumulation of mitochondria in the perinuclear region, and knockdown of both hMiro1 and hMiro2 further enhanced this effect (Figure 6B). Fluorescence from the immunostaining of Tom20 (an OMM marker) but not HSP60 (a matrix marker) in hMiro1 and hMiro2 double knockdown cells was noticeably weaker than that in control siRNA treated cells at 3 h after CCCP treatment (Figure 6C), supporting the notion that hMiro knockdown facilitated an early event in Parkin-mediated mitophagy.

### The conserved Ser-156 residue in hMiro1 is not required for PINK1/Parkin-mediated degradation under normal or CCCP treatment conditions in HeLa cells

**Figure 7:**
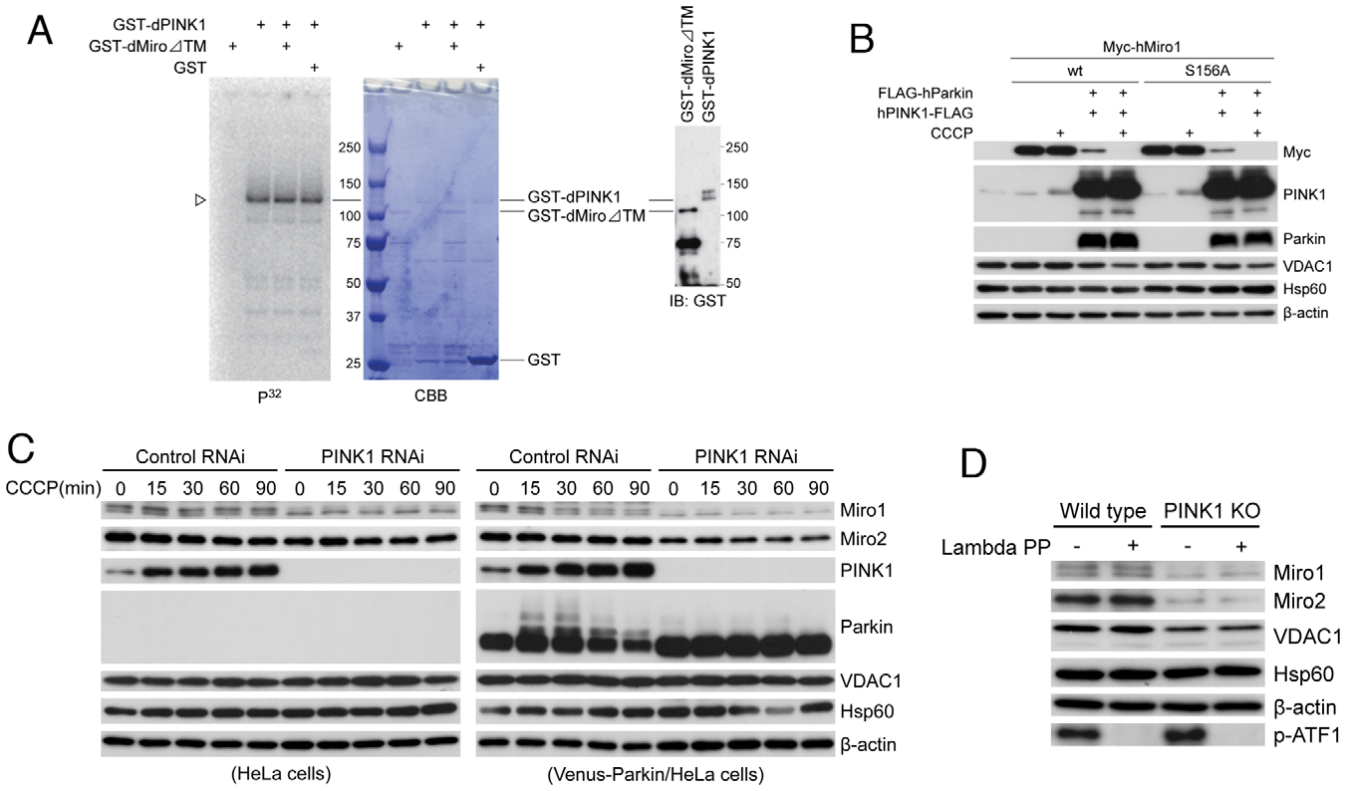
Effects of PINK1 loss of function on endogenous Miro protein level in mammalian cells and testing potential direct phosphorylation of Miro by PINK1. (A) Lack of direct phosphorylation of GST-dMiroΔTM by 2× GST-dPINK1. Autoradiography (P^32^) and Coomassie Brilliant Blue (CBB) staining of the same SDS-PAGE gel of the *in vitro* kinase reactions were shown. The presence of the GST-dMiroΔTM and 2× GST-dPINK1 proteins was further confirmed by Western blot with anti-GST. The detected P^32^ signals represent PINK1 autophosphorylation. (B) Western blot analysis comparing the stability of transfected Myc-hMiro1-wild type (wt) and Myc-hMiro1-S156A in HeLa cells with or without CCCP treatment and in the presence or absence of hPINK1+hParkin co-expression. (C) Western blot analysis of HeLa cells or Venus-Parkin stably transfected HeLa cells with or without CCCP treatment under control RNAi or PINK1 RNAi conditions. (D) Western blot analysis of mouse Miro1 and Miro2 levels in wild type or PINK1 knockout (KO) MEF cells. Extracts were treated with or without lambda phosphatase before Western blot analysis. Note that whereas the signals for a control phsopho-ATF1 (p-ATF1) protein disappeared after lambda phosphatase treatment, neither Miro1 nor Miro 2 showed mobility or intensity change after the same treatment.

We further investigated the molecular mechanisms by which the PINK1/Parkin pathway regulates Miro protein level or stability. While our paper was under review, a report showed that PINK1 phosphorylates Miro at a conserved S156 residue, and that this phosphorylation activates proteasomal degradation of Miro in a Parkin-dependent manner [34]. However, repeated *in vitro* kinase assays using an active GST-dPINK1 recombinant protein capable of efficient autophosphorylation [42] failed to show phosphorylation of GST-dMiroΔTM, a GST fusion protein of full-length dMiro with the transmembrane domain deleted (Figure 7A). *Drosophila* or mammalian PINK1 protein affinity purified from HEK293 cells by immunoprecipitation also failed to phosphorylate GST-dMiroΔTM in our assays (data not shown).

To further probe the significance of S156 phosphorylation in facilitating the proteasomal degradation of Miro promoted by the PINK1/Parkin pathway, we introduced S156A mutations into hMiro1 or hMiro2 and examined the stability of the mutant proteins in HeLa cells co-transfected with PINK1 and Parkin, under normal or CCCP treatment conditions. As shown in Figure 7B, the wild type and S156A mutant forms of hMiro1 were equally susceptible to PINK1/Parkin- mediated degradation under both conditions. The wild type and S156A mutant forms of hMiro2 behaved similarly as well (data not shown). Thus, the PINK1/Parkin pathway may regulate Miro protein level independent of S156 site phosphorylation under these experimental conditions in HeLa cells.

### Effect of loss of PINK1 function on Miro protein level in mammalian cells

We also examined the effect of loss of PINK1 function on Miro protein level in mammalian cells. For this purpose, we used both HeLa cells with PINK1 knockdown and MEF cells derived from PINK1 (−/−) knockout mice. Surprisingly, unlike the situation in *Drosophila*, endogenous Miro1 or Miro2 protein levels were significantly reduced in PINK1 RNAi cells under normal or CCCP treatment conditions (Figure 7C, PINK1 RNAi in HeLa cells or Venus-Parkin stably transfected HeLa cells) and in PINK1 (−/−) MEF cells (Figure 7D). The introduction of Venus-Parkin resulted in CCCP/PINK1-dependent degradation of Miro1 (Figure 7C, Control RNAi in Venus-Parkin transfected HeLa cells). Thus, loss of PINK1 function in mammalian cells can lead to reduced expression of Miro1 and Miro2 proteins, presumably through mechanisms distinct from that operating under PINK1/Parkin co-overexpression condition.

## Discussion

Mitochondrial dysfunction has long been implicated in the pathogenesis of PD. However, the exact mechanisms by which mitochondrial dysfunction arises in the disease process and how cells, particularly neurons, handle dysfunctional mitochondrial are not well understood. The identification of a mitochondrial quality control system involving two FPD genes, PINK1 and Parkin, has provided a much-needed point of entry to elucidate the role of mitochondria in the pathogenesis of PD. Here we showed that PINK1 directly regulates mitochondrial transport and that it affects the stability and/or activity of Miro, a mitochondrial Rho GTPase with a well-establish function in mitochondrial transport. Our conclusion is supported by the following evidence: 1) dMiro protein level is negatively regulated by PINK1 and Parkin *in vivo* in *Drosophila*; 2) Overexpressed PINK1 and Parkin act together to promote the ubiquitination and degradation of hMiro1 in HeLa cells; 3) Reduction of the activities of Miro or other components of the mitochondrial transport machinery effectively rescued *dPINK1* mutant phenotypes. 4) Overexpression of dMiro in DA neurons phenocopied *dPINK1* loss-of-function effects; 5) Manipulation of dPINK1 activity produced clear mitochondrial motility phenotypes opposite to that observed for dMiro manipulation in *Drosophila* larval motor neurons. Together, these results support that the mitochondrial transport defects caused by PINK1 inactivation represent one of the key pathogenic events that contribute to PD pathogenesis in the *Drosophila* model.

Neurons are highly polarized cells that rely heavily on axonal transport to distribute to axons and synapses critical proteins and organelles synthesized in the cell body, thereby maintaining neuronal function and health. Defects in axonal transport are often linked to diseases affecting peripheral neurons that tend to extend very long axons [21], [22], [29]. Although the symptoms of PD patients mainly arise from the loss of DA neurons, some PD patients carrying particular *PINK1* and *Parkin* mutations developed peripheral neuropathy with unknown cause [18]–[20]. Our results showing the PINK1/Parkin pathway playing a critical role in regulating mitochondrial transport offers one potential explanation of the peripheral neuropathy symptoms observed in these PINK1 or Parkin-linked PD cases. It would be interesting to examine whether Miro protein level or activity is affected by these particular mutations in human cells. Moreover, we propose that defects in PINK1/Parkin-regulated mitochondrial transport may offer one explanation of the selective vulnerability of DA neurons observed in PD patients and animal models. DA neurons that make elaborate and long projections may be particularly vulnerable to impairment of the mitochondrial transport system.

Our results offer new insights into the mode of action of the PINK1/Parkin pathway in mitochondria quality control. We showed that, in *Drosophila* models, PINK1 OE led to decreased mitochondrial flux and net velocity, as observed in Miro knockdown background. In addition, we found that Miro knockdown could facilitate an early step of mitophagy in mammalian cells. These observations, together with the finding that the normally labile PINK1 protein is stabilized on damaged mitochondria [39], suggest a scenario whereby the accumulation of PINK1 on damaged mitochondria and the subsequent turnover of Miro could exert neuroprotection by (1) preventing damaged mitochondria from being anterogradely transported along the axons, thus increasing their chance of getting eliminated in the soma; and (2) promoting elimination of damaged mitochondria through mitophagy. This potentially explains the normal protective function of PINK1. When PINK1 function is impaired, however, on one hand mitochondria become dysfunctional as evidenced by morphology changes and impaired electron transport chain function [3]–[5], [33], [43]–[45], on the other hand, the anterograde mitochondrial transport is enhanced as shown in this study in *Drosophila* models. As a result, the dysfunctional mitochondria would have increased retention in the axons and synapses, resulting in increased reactive oxygen species (ROS) production, oxidative damage, and subsequent synaptic and axonal degeneration and eventual neuronal loss, at least in the *Drosophila* models. Many details of this model await further experimental validation. For example, it has been suggested that the reported effect of PINK1/Parkin on mitochondrial autophagy may not operate in the same manner in primary neurons as compared to cultured non-neuronal cells [46]. It also remains to be determined whether the effects of Miro on mitochondrial transport and mitophagy reflect a functional antagonism between these two processes, or two distinct functions of Miro in neuronal maintenance. In this respect, it is worth noting that the effect of Miro overexpression on cell survival in *Drosophila* is cell type-dependent: it causes DA neuron loss but has no obvious effect on muscle integrity (data not shown). It is possible that different tissues may have different sensitivities to impairments of Miro function. For example, muscle cells may be less susceptible to mitochondrial transport defects than neurons.

One interesting difference we observed between *Drosophila* and mouse systems was that although activation of the PINK1/Parkin pathway led to reduced Miro protein level in both systems, the loss of PINK1 in *Drosophila* resulted in increased steady-state Miro protein level, whereas its loss in mammalian cells as in *PINK1 (−/−)* MEF cells or PINK1 RNAi HeLa cells had the opposite effect. The mechanism of Miro downregulation in PINK1 loss-of-function mammalian cells is currently unknown, but it is presumably different from that used by activation of the PINK1/Parkin pathway. Since the upregulation of dMiro in *dPINK1* mutant background is likely causal to DA neuron degeneration, as indicated by the rescue of DA neuron loss in *dPINK1* mutant by dMiro-RNAi and the induction of DA neuron loss by dMiro-OE alone, it is tempting to speculate that the downregulation of Miro levels in *PINK1 (−/−)* mouse, as opposed to the dMiro upregulation in *Drosophila PINK1* mutant, might contribute to the lack of DA neuron degeneration phenotype in the mouse PINK1 models [47]–[50]. Testing this hypothesis will require *in vivo* studies boosting Miro expression levels in wild type and *PINK1 (−/−)* mouse.

Our results also provide new insights into the process by which the PINK1/Parkin pathway promotes mitophagy. Previous studies suggested that upon recruitment to damaged mitochondria, Parkin activates the ubiquitin proteasome system to effect wide-spread degradation of OMM proteins in an autophagy-independent manner, and it was further proposed that this remodeling of OMM is important for a subsequent step of mitophagy [40]. The previously identified Parkin substrates, Mfn1 and Mfn2, although important for the effect of Parkin on mitochondrial fission/fusion dynamics, are not necessary for Parkin-induced mitophagy [40], [51]. Here we show that removal of Miro by the PINK1/Parkin pathway, in a presumably autophagy-independent but ubiquitination-dependent manner, facilitated mitophagy. Interestingly, knockdown of mammalian Miro itself promotes the formation of ring-like or round-shaped mitochondrial morphology, which is often observed in depolarized, mitophagy-ready mitochondria (Figure 6B; [13]). It is possible that the removal of Miro from OMM exposes certain recognition signals for the autophagy machinery, or that Miro/Milton/Kinesin-mediated mitochondrial transport may normally antagonize the mitophagy process. Supporting the latter scenario, an interaction between the BECLIN 1-interacting protein AMBRA1 and the dynein motor complex has been implicated in mammalian autophagy [52]. It also remains to be understood at the mechanistic level how PINK1 cooperates with Parkin to promote the ubiquitination and degradation of Miro. One attractive hypothesis is that PINK1 may directly phosphorylate Miro to promote its subsequent ubiquitination and degradation by Parkin, as suggested by a recent study [34]. However, our biochemical data have so far failed to support this hypothesis. It is possible that the divergent results are due to the different cell lines used or other experimental conditions. Alternatively, PINK1 may directly act on Parkin to promote Parkin's mitochondrial recruitment or activity in activating ubiquitin proteasome system-mediated ubiquitination and degradation of Miro. Further studies are needed to elucidate the molecular mechanisms of PINK1/Parkin action.

Finally, it is worth mentioning that studies in *Drosophila* models have identified a number of genetic modifiers of PINK1/Parkin [35], [53], [54]. While some of these genetic modifier genes may be directly related to the seemingly diverse biological activities of the PINK1/Parkin pathway, possibly mediated by distinct PINK1/Parkin substrates, others may reflect cellular compensatory responses to cope with the mitochondrial dysfunction caused by PINK1/Parkin inactivation. The fact that manipulations of each of these different cellular processes exert clear functional rescue of PINK1/Parkin mutant phenotypes suggests that there exists a signaling network linking the diverse activities of PINK1/Parkin in mitochondria biology with the nuclear-encoded cellular responses to mitochondrial dysfunction, and that many key players in this network represent novel and rational therapeutic targets.

## Materials and Methods

### Fly strains and reagents

Flies were raised according to standard procedures at indicated temperatures. Sources of fly strains and other reagents are as follows: *dPINK1^B9^*: Dr. J. Chung [3]; *UAS-dMiro* and anti-dMiro antibody: Dr. K. Zinsmaier [31]; *TH-GAL4*, *UAS-PINK1*, *UAS-PINK1 RNAi* and rabbit anti-*Drosophila* TH antibody: described before [5]; *UAS-Miro RNAi^106683^*: Vienna *Drosophila* RNAi Center; *UAS-Miro RNAi^27695^*, *UAS-Milton RNAi^28385^* and *UAS-Khc RNAi^25898^*: Harvard Transgenic RNAi Project (TRiP) and Bloomington *Drosophila* Stock Center; all other fly lines: Bloomington *Drosophila* Stock Center; FLAG-hParkin mutants and HA-ubiquitin: Drs. N. Matsuda, K. Tanaka and S. Hatakeyama; Myc-hMiro1 and Myc-hMiro2 plasmids: Dr. P. Aspenström [30]; hPINK1 cDNAs were cloned into pcDNA3-FLAG vector.

Antibodies used in this study are as follows: anti-RHOT1/Miro1 (4H4, Abnova), anti-RHOT2/Miro2 (Protein technology Group), anti-PINK1 (Novus), anti-Parkin (PRK8, Santa Cruz Biotechnology), anti-Tom20 (FL-145, Santa Cruz Biotechnology), anti-VDAC1 (Abcam), anti-OXPHOS Complex IV subunit I/COX I (Invitrogen), anti-Tim23 (BD), anti-NDUFA9 (Invitrogen), anti-Cytochrome *c* (BD), anti-HtrA2/Omi (as described in [55]), anti-Hsp60 (BD), anti-PDHA1 (Abcam), anti-HA (3F10, Roche), anti-Myc (4A6, Millipore; #2272, Cell Signaling Technology), anti-β-actin (AC-15, Sigma-Aldrich), anti-α-tubulin (DM1A, Millipore), Peroxidase anti-Guinea Pig IgG antibody (Jackson ImmunoResearch), Texas Red-conjugated anti-HRP (Jackson ImmunoResearch), Alexa Fluor 488 nm-conjugated goat anti-chicken IgG (Invitrogen) and Alexa Fluor 594 nm-conjugated goat anti-rabbit IgG (Invitrogen).

### Fly wing posture, behavior, ATP measurement, and immunohistochemistry

These assays were carried out essentially as described before [35]. The thoracic ATP level was measured using a luciferase based bioluminescence assay (ATP Bioluminescence Assay Kit HS II, Roche applied science) as described [35].

Whole-mount brain immunohistochemistry for TH and mitoGFP was performed as described previously [35]. For DA neuron mitochondrial morphology analysis, mitoGFP was expressed in *Drosophila* DA neurons using the *TH-Gal4* driver. Brains from 3-day-old adult flies of the indicated genotypes were immunostained with the anti-TH antibody to label DA neuron and anti-GFP antibody to label mitochondria. For measurement of mitochondrial size distribution, the size of each mitochondrial aggregate was represented by the length of its longest axis. The percentage of DA neurons in the PPL1 cluster that have one or more mitochondria exceeding the indicated size was shown. Six flies of each genotype were used for the analysis.

### Live imaging of mitochondrial movement

The motor neuron-specific *OK6-Gal4* driver was used to express *UAS-mitoGFP* in the larval segment neurons, and 3^rd^ instar male larvae raised at 29°C were used for live imaging. Larvae were briefly washed with water and anesthetized for 4 min in 1.5 ml eppendorf tubes containing 4 µl suprane (Baxter International Inc.), before being placed ventral side up into a small chamber. The chamber was created on glass slide with double-sided tape and cover glass. Additional suprane (4 µl) was introduced into the chamber before it was sealed with Valap (1∶1∶1 amount of vaseline, lanolin, parafin wax). Mitochondria were viewed with an upright Leica DM6000 B microscope equipped with a laser scanner and a 63× oil-immersion objective. Larvae were positioned to have their ventral ganglion (VG) appearing on the right of the acquired image and segmental nerves aligned horizontally across the image. Before recording mitochondrial movement, a centered region of 1024×200 pixel (61.5 µm×12.0 µm, 4× digital zoom) close to the VG was photobleached for 30 s with 488 nm excitation argon laser set at 80% output power. The viewing field was then zoomed out (2.5× digital zoom) and mitochondrial movement was immediately recorded by time-lapse video (2 s/frame, 300 s total) in a region of 1024×150 pixel (98.4 µm×14.4 µm) with the laser power reduced to 10% of the maximum output. The pinhole was set at 200 µm for all the experiments. Time-lapse images were acquired within 30 min of anesthetization. Mitochondrial flux was calculated by normalizing the number of mitochondria that passes certain point within the time frame examined. Mitochondrial net velocity was calculated by normalizing mitochondrial net displacement with time. At least 5 larvae of each genotype were analyzed.

### Cell culture and transfection

HeLa cells were maintained at 37°C with 5% CO2 atmosphere in DMEM (Wako) supplemented with 10% FCS (GIBCO) and non-essential amino acids (Invitrogen). Plasmids and siRNA duplexes (Invitrogen) were transfected using Lipofectamine 2000 (Invitrogen) and Lipofectamine RNAiMAX (Invitrogen), respectively, according to manufacturer's instructions. To depolarize the mitochondria, HeLa cells were treated with 10 µM CCCP (Sigma-Aldrich) at 36 hr (for plasmids) or 72 hr (for siRNA) post-transfection.

The S156A mutations were introduced into human Miro1 and Miro2 by PCR-based mutagenesis using the following primers: For hMiro1, Forward primer: 5′- **GCA** gag ctcttttatt acgcac -3′; Reverse primer: 5′- tatg ttcttcaggt ttttcgc -3′. For hMiro2, Forward primer: 5′- **GCA** gagct gttctactac gc -3′; Reverse primer: 5′- ga tgttcctcag gttcttggc -3′. Full-length sequences of hMiro1 S156A and hMiro2 S156A were confirmed by sequencing.

### Immunoblotting, immunoprecipitation, and *in vitro* phosphorylation

For brain extract preparation, 6 fly heads were quickly dissected and homogenized in 60 µl SDS-PAGE sample buffer. 5 µl brain extracts from each genotype were loaded onto SDS-PAGE gel. Guinea pig anti-dMiro antibody (1∶20000, from Dr. K. Zinsmaier) and Peroxidase Anti-Guinea Pig IgG antibody (1∶10000, Jackson ImmunoResearch Labs) were used for Western Blot. For HeLa cell-based biochemical analysis, cells were lysed in 1% Triton X-100 -based lysis buffer (10 mM Tris-HCl [pH 7.6], 120 mM NaCl, 5 mM EDTA, 1% Triton X-100 and protease inhibitor [Nacalai Tesuque]). Immunoprecipitation was performed using Immunoprecipitation Kit-Dynabeads Protein G (Invitrogen) according to manufacturer's instructions.

*In vitro* kinase assay was performed essentially as described [42], using a 2× GST-dPINK1 fusion protein with GST fused at both the N- and C-terminus of dPINK1 as the kinase source and a GST-dMiroΔTM fusion protein as the substrate. GST-dMiroΔTM covers amino acids 1–634 of the full-length dMiro protein. The GST-dMiro-ΔTM plasmid was constructed by amplifying a Myc-tagged dMiro fragment without the transmembrane domain from a *pUAST-Myc-dMiro* plasmid (obtained from Dr. K. Zinsmaier) using 5′-CGCCCG-GGTGAGCAGAAACTCATCTCTGAAGAAG-3′ and 5′-ATGCGGCCGCTACTTGGG-GTCCTCCGTC-ATC-3′ as primers. The amplified fragment was inserted into the *SmaI* and *NotI* cloning sites of the *pGEX-6P-1* vector. Recombinant GST fusion proteins were purified from bacteria according to standard protocols.

### Immunocytochemistry

Cells were fixed with 4% paraformaldehyde in PBS and permeabilized with 0.2% Triton X-100 (for mitophagy in Figure 6C) or 0.5% Triton X-100 (for mitochondrial morphology in Figure 6B) in PBS. Cells stained with the appropriated antibodies and counterstained with DAPI were imaged using a laser-scanning microscope (LMS510 META; Carl Zeiss, Inc.) with a Plan-Apochromat 63×NA1.4 or 100×/1.4 Oil differential interference contrast objective lens. Image contrast and brightness were adjusted in Image Browser (Carl Zeiss, Inc.)

### Statistical analysis

Two-tailed Student's *t* tests were used for statistical analysis. *p* values of <0.05, <0.01, and <0.005 were indicated with one, two, and three asterisks (*), respectively.

## Supporting Information

Figure S1 hMiro2 on damaged mitochondria is ubiquitinated by PINK1 and Parkin. (a) HeLa cells transfected with the indicated plasmids were treated with or without 10 µM CCCP for 90 min. Immunoprecipitate of hMiro2 was analyzed for poly-ubiquitination modification and co-precipitation of PINK1 and Parkin. Arrowheads, co-precipitated Parkin or PINK1 with hMiro2; Asterisks, oligo-ubiquitinated hMiro2 detected by anti-Myc antibody. (b) Pathogenic mutant forms of PINK1 or Parkin abolished hMiro2 ubiquitination. The ubiquitination assay was performed as in (a). The Parkin K211N mutation in the linker domain is reported to abolish both its E3 ubiqutin-ligase activity and its ability to translocate to damaged mitochondria. The Parkin T415 mutation in the RNIG2 domain disrupts its E3 activity, while partially retaining its mitochondrial translocation ability [56]. The PINK1 L347P mutant is reported to have reduced kinase activity [57].
(TIF)

Video S1 Mitochondrial movement in the axons of wild-type *Drosophila* larval segment neurons. Representative time-lapse video of mitochondrial movement in a segment neuron of wild-type *Drosophila* larva is shown. Mitochondrial movement was visualized by the mitoGFP fluorescence. Mitochondrial movement to the left represents anterograde axonal transport. The video was acquired at a speed of 2 sec/frame and played at 4 frames/sec (1 s movie time = 8 s real time).
(AVI)

Video S2 PINK1-OE arrests mitochondrial movement in the axons of *Drosophila* larval segment neurons. Representative time-lapse video of mitochondrial movement in a segment neuron of PINK1-OE *Drosophila* larva is shown. Mitochondrial movement was visualized by the mitoGFP fluorescence. PINK1-OE decreases mitochondrial length and flux in both anterograde and retrograde directions. Mitochondrial movement to the left represents anterograde axonal transport. The video was acquired at a speed of 2 sec/frame and played at 4 frames/sec (1 s movie time = 8 s real time).
(AVI)

Video S3 PINK1-RNAi promotes anterograde mitochondrial movement in a *Drosophila* larval segment neuron axon. Representative time-lapse video of mitochondrial movement in a segment neuron of PINK1-RNAi *Drosophila* larva is shown. Mitochondrial movement was visualized by the mitoGFP fluorescence. PINK1-RNAi increases mitochondrial length and anterograde mitochondrial flux and movement speed. Mitochondrial movement to the left represents anterograde axonal transport. The video was acquired at a speed of 2 sec/frame and played at 4 frames/sec (1 s movie time = 8 s real time).
(AVI)

Video S4 Miro-RNAi arrests mitochondrial movement in the axons of *Drosophila* larval segment neurons. Representative time-lapse video of mitochondrial movement in a segment neuron of Miro-RNAi *Drosophila* larva is shown. Mitochondrial movement was visualized by the mitoGFP fluorescence. Miro-RNAi decreases mitochondrial length and flux in both anterograde and retrograde directions. Mitochondrial movement to the left represents anterograde axonal transport. The video was acquired at a speed of 2 sec/frame and played at 4 frames/sec (1 s movie time = 8 s real time).
(AVI)

Video S5 Miro-OE increases mitochondrial movement speed in the axons of *Drosophila* larval segment neurons. Representative time-lapse video of mitochondrial movement in a segment neuron of Miro-OE *Drosophila* larva is shown. Mitochondrial movement was visualized by the mitoGFP fluorescence. Miro-OE increases mitochondrial length and anterograde movement speed. Mitochondrial movement to the left represents anterograde axonal transport. The video was acquired at a speed of 2 sec/frame and played at 4 frames/sec (1 s movie time = 8 s real time).
(AVI)

Video S6 Fis1-OE promotes mitochondrial flux in the axons of *Drosophila* larval segment neurons. Representative time-lapse video of mitochondrial movement in a segment neuron of Fis1-OE *Drosophila* larva is shown. Mitochondrial movement was visualized by the mitoGFP fluorescence. Overexpression of the mitochondrial fission protein Fis1 (FIs1-OE) decreases mitochondrial length but increases mitochondrial flux in both anterograde and retrograde directions. Mitochondrial movement to the left represents anterograde axonal transport. The video was acquired at a speed of 2 sec/frame and played at 4 frames/sec (1 s movie time = 8 s real time).
(AVI)

Video S7 Marf-RNAi promotes mitochondrial flux in the axons of *Drosophila* larval segment neurons. Representative time-lapse video of mitochondrial movement in a segment neuron of Marf-RNAi *Drosophila* larva is shown. Mitochondrial movement was visualized by the mitoGFP fluorescence. Knockdown of the mitochondrial fusion protein Marf (Marf-RNAi) decreases mitochondrial length but increases mitochondrial flux in both anterograde and retrograde directions. Mitochondrial movement to the left represents anterograde axonal transport. The video was acquired at a speed of 2 sec/frame and played at 4 frames/sec (1 s movie time = 8 s real time).
(AVI)
