# Supplementary figures and images for: Parkinson's Disease–Associated Kinase PINK1 Regulates Miro Protein Level and Axonal Transport of Mitochondria
Source: PLoS Genet. 2012 Mar 1;8(3):e1002537. doi: 10.1371/journal.pgen.1002537 (PMC3291531; doi:10.1371/journal.pgen.1002537)

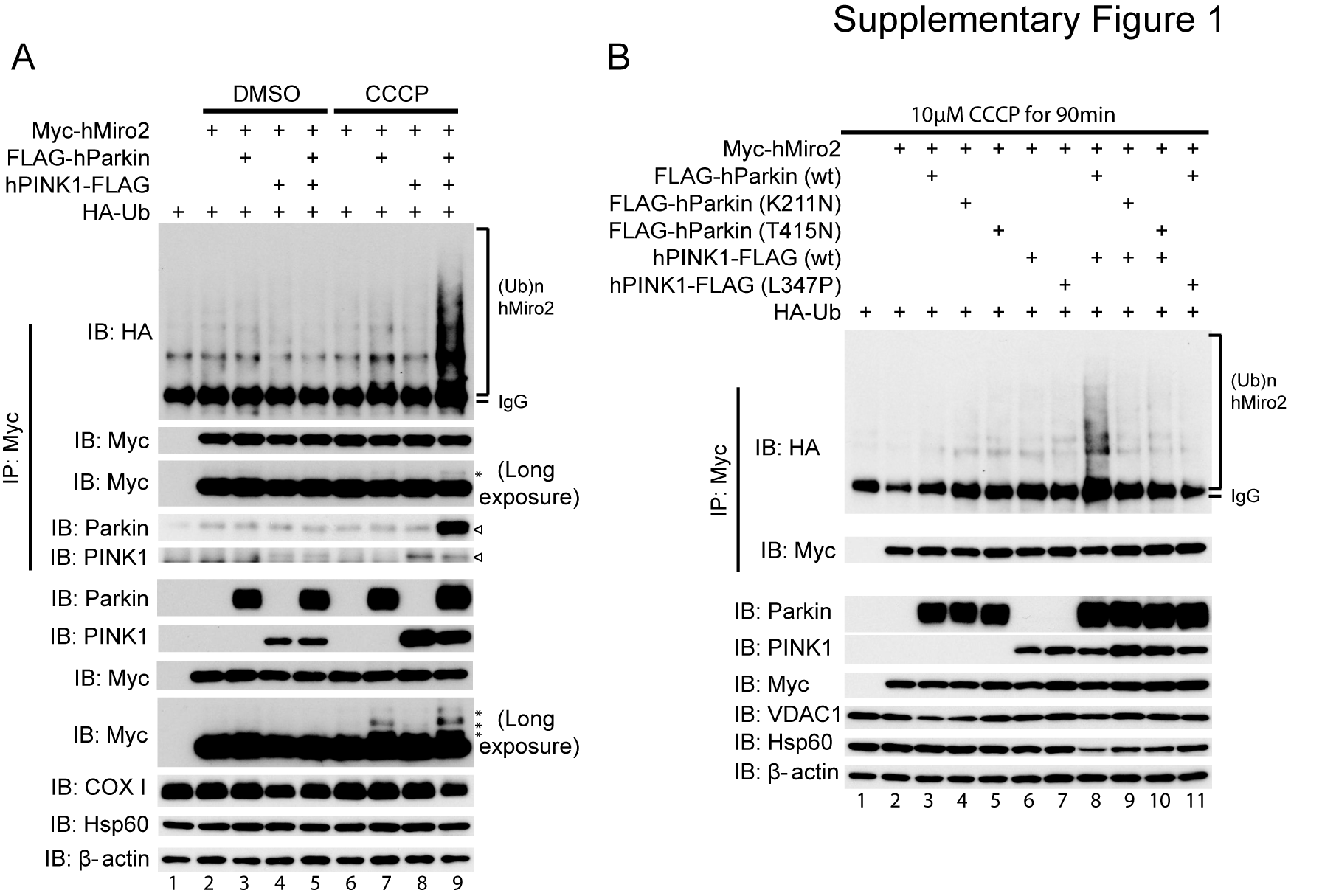

Supplement: Figure S1 — hMiro2 on damaged mitochondria is ubiquitinated by PINK1 and Parkin. (a) HeLa cells transfected with the indicated plasmids were treated with or without 10 µM CCCP for 90 min. Immunoprecipitate of hMiro2 was analyzed for poly-ubiquitination modification and co-precipitation of PINK1 and Parkin. Arrowheads, co-precipitated Parkin or PINK1 with hMiro2; Asterisks, oligo-ubiquitinated hMiro2 detected by anti-Myc antibody. (b) Pathogenic mutant forms of PINK1 or Parkin abolished hMiro2 ubiquitination. The ubiquitination assay was performed as in (a). The Parkin K211N mutation in the linker domain is reported to abolish both its E3 ubiqutin-ligase activity and its ability to translocate to damaged mitochondria. The Parkin T415 mutation in the RNIG2 domain disrupts its E3 activity, while partially retaining its mitochondrial translocation ability [56]. The PINK1 L347P mutant is reported to have reduced kinase activity [57]. (TIF) [file pgen.1002537.s001.tif]
